# The Consequences of Female Genital Mutilation on Psycho-Social Well-Being: A Systematic Review of Qualitative Research

**DOI:** 10.1177/10497323211001862

**Published:** 2021-06-08

**Authors:** Sarah O’Neill, Christina Pallitto

**Affiliations:** 1 Université Libre de Bruxelles, Brussels, Belgium; 2 UNDP-UNFPA-UNICEF-WHO-World Bank Special Programme of Research, Development and Research Training in Human Reproduction (HRP), World Health Organization, Geneva, Switzerland

**Keywords:** female genital mutilation, FGM, Female Genital Cutting, FGC, psycho-social well-being, qualitative data synthesis, systematic review, health-seeking behavior, stigma, marriageability

## Abstract

The health consequences of female genital mutilation (FGM) have been described previously; however, evidence of the social consequences is more intangible. To date, few systematic reviews have addressed the impact of the practice on psycho-social well-being, and there is limited understanding of what these consequences might consist. To complement knowledge on the known health consequences, this article systematically reviewed qualitative evidence of the psycho-social impact of FGM in countries where it is originally practiced (Africa, the Middle East, and Asia) and in countries of the diaspora. Twenty-three qualitative studies describing the psycho-social impact of FGM on women’s lives were selected after screening. This review provides a framework for understanding the less visible ways in which women and girls with FGM experience adverse effects that may affect their sense of identity, their self-esteem, and well-being as well as their participation in society.

## Introduction

Female genital mutilation (FGM) includes procedures that intentionally alter or cause injury to the female genital organs for nonmedical reasons (WHO, 2016, 2018a). It is estimated that more than 200 million women and girls alive today have undergone some form of FGM (United Nations International Children’s Emergency Fund [UNICEF], 2016). The complex reasons for which the practice is performed and its meaning within the social context have also been largely discussed (Boddy, 1989; Gruenbaum, 2001; Shell-Duncan & Hernlund, 2000; Shell-Duncan et al., 2011). Commonly mentioned reasons for practising are linked to various forms of control of female sexuality—such as to prevent debauchery and promiscuity, and to ensure virginity before marriage and marital fidelity (Boddy, 1989; Fahmy et al., 2010; Hosken, 1993; O’Neill, 2018; Shell-Duncan & Hernlund, 2000); due to conceptions of purity and aesthetics (Boddy, 1989; Fahmy et al., 2010; O’Neill, 2018); as a way to mark coming of age and status change within the community (Ahmadu, 2000, 2007; Leonard, 2000); and for religious reasons due to the belief that the practice is a religious recommendation (Abu-Salieh, 2001; Isa et al., 1999; Johnson, 2000; Merli, 2010) even though FGM is not formally prescribed by any religion.

The World Health Organization classifies FGM into 4 major types. Type 1 is the partial or total removal of the clitoral glans and/or the prepuce. Type 2 is the partial or total removal of the clitoral glans and the labia minora, with or without removal of the labia majora. Type 3, also known as infibulation, is the narrowing of the vaginal opening through the creation of a covering seal. The seal is formed by cutting and repositioning the labia minora, or labia majora, sometimes through stitching, with or without removal of the clitoral prepuce and glans. Type 4 includes all other harmful procedures to the female genitalia for nonmedical purposes, for example, pricking, piercing, incising, scraping, and cauterizing the genital area.

FGM has been shown to have negative effects on health and other aspects of well-being. Previous reviews and meta-analyses have described the physical and nonphysical health consequences of FGM, with some reviews calculating pooled estimates of risk by comparing women exposed to FGM with women not exposed to FGM (Berg & Underland, 2012; Kimani et al., 2016). Two other reviews explored the socio-economic, psychological, and sexual consequences of the practice, but these systematic reviews did not conduct quantitative analyses (Berg et al., 2010; Mpinga et al., 2016). To complement previous reviews and to add to the knowledge about long-term impacts of FGM in different settings, this review synthesizes the qualitative evidence of psycho-social impacts of FGM on multiple aspects of women’s lives, showing the wide range of ways that FGM affects women and how they interact with family, community members, and society more generally. Defining well-being is an important step in understanding how FGM can adversely affect women’s psycho-social health and how they engage with others in their social environment. The concept of well-being and related measures has not been consistently operationalized in research on FGM.

### Well-Being and Social Norms—A Definition

The World Health Organization (WHO, 2018b) describes mental health and well-being as “every individual being able to realize his or her own potential, to cope with the normal stresses of life, to work productively and fruitfully, and to be able to make a contribution to her or his community.” This definition includes the eudaimonic concept of well-being, which explains that positive psychological functioning and actualization of one’s human potential are key components in achieving well-being (Rogers, 1961; Ryff, 1989; Ryff & Keyes, 1995). While “subjective well-being” is frequently used interchangeably with the term “happiness,” which is thought to be linked to personal factors, social-environmental factors, and cultural factors (Deci & Ryan, 2008), the subjective feeling of being happy does not necessarily mean that a person is psychologically well (Deci & Ryan, 2008). Recognizing the complexity of defining and measuring the constructs of well-being is beyond the scope of this review, measurable concepts of well-being were identified in the literature and summarized in this article. These include self-esteem, stigma, relationship factors, psychological health, social inclusion/exclusion, interpersonal and societal interactions and relationships, and well as health seeking.

It has been widely accepted that FGM is a social norm (Mackie et al., 2014; Shell-Duncan et al., 2011; Shell-Duncan & Hernlund, 2000; UNICEF, 2013). A practice is considered a social norm in a particular context if it meets the following conditions: a rule of behavior exists around the practice; individuals are aware of the rule and believe that it applies to them; and individuals comply with this rule to conform to social conventions among their common ethnic, religious, or social group and to avoid social sanctions (UNICEF, 2013). Nonconformity to social norms is commonly sanctioned to various degrees with consequences ranging from personal feelings of guilt to social exclusion, stigma, and shame (Goffman, 1963). Therefore, there is a strong disincentive to deviate from social norms and fear of sanctions are a strong driver to comply with them. Social norms are considered culturally defined within a particular cultural context (Edberg & Krieger, 2020) and are central to our understanding of how communities maintain and transmit dominant social practice (Ortner, 1984). Social norm change can be a fluid process during which the relationship between any given norm and the deeper structures of meaning, power, and social organization also evolve. Changes in these structures may occur, yet the norm that justifies these structures remain because they are ingrained as habit or tradition; or the norm may change, but the deeper social structure does not (Edberg & Krieger, 2020). The ways social norms evolve among subgroups in migrant or diaspora populations is an important consideration, but beyond the scope of this article, which is more focused on the differential impact of social norms on individuals when they are discordant from their general population.

To our knowledge, it is the first review and synthesis of qualitative research on the psycho-social consequences of FGM, providing a personal perspective of the women who have undergone this practice. The review applies the social ecological framework (McLeroy et al., 1988) to describe and classify the ways that women’s lives are affected by FGM. Recognizing that the effects of FGM will differ greatly depending on one’s social context, this review analyses separately those studies conducted in settings where FGM is widely practiced and socially normative (high prevalence) from those studies conducted in settings where the practice is less common but exists among some population subgroups, such as among immigrant communities in settings where it is not a normative practice.

## Methods

### Search Strategy

A systematic review of the literature was conducted to identify all potential consequences of FGM beyond health consequences. Broad areas of interest were education, employment, fertility, honor, marriage, and other psycho-social outcomes affecting individual women. The search was undertaken in online databases that capture peer-reviewed public health and social science literature as well as “gray literature” such as dissertations and unpublished reports. The review follows the PRISMA guidelines and the protocol is registered at PROSPERO.ac.uk. The search was undertaken on the databases Pubmed, ISI Web of knowledge, and SCOPUS/Science direct. OCLC World CAT, OAIster, and Google scholar and hand searches were also performed to search for additional studies and grey literature.

The first search was performed for the time-period of January 1, 1990 to August 1, 2017. The search was updated to include studies up until 29 October 2018. Duplicates were deleted. Titles and abstracts were screened by both authors. The search terms used for title and abstract searches are listed in the Supplemental File.

### Study Selection

Included were any studies in English language that explored the psycho-social consequences of FGM, using clearly described qualitative methods, such as interviews, focus group discussions, and participant observation (ethnography). Narratives, commentaries, case reports, and discursive philosophical pieces with no clearly described methodology and data collection were excluded. Studies that assessed psycho-social impact using quantitative methods were reviewed separately. The population included (1) women and men from FGM practising communities living in practicing countries; (2) women and men from FGM practising communities residing in high-income diaspora settings (low-FGM prevalence setting); excluded were studies addressing male circumcision and female genital cosmetic surgery. Following the GRADE-CERQual approach (Lewin et al., 2018), studies were assessed for criteria that inform the confidence in the findings. These include methodological limitations, coherence, adequacy of data, and relevance. Studies that did not satisfy at least three of the four CERQual criteria were excluded from the analysis.

After the first round of screening, a full-text assessment was undertaken on 486 papers to assess relevance and to confirm compliance with GRADE-CERQual criteria. Included studies were thematically coded based on the themes included in the search (i.e., education, employment, fertility, honor, marriage, and other psycho-social outcomes affecting individual women) as well as emerging themes (stigma in diaspora; stigma in country of origin; anxiety, vulnerability, and psycho-social issues; experiences in health care settings; relationships with men; intimacy; divorce; relationships with mother/family members; and relationships with wider community).

## Results

### Screening Results

During the first round of screening from January 1, 1990 to August 1, 2017, a total of 2,288 results were obtained using Pubmed, Scopus, and ISI Web of Knowledge. Five additional documents were identified and included for review. The second screening covered the period from August 2, 2017 to October 29, 2018, and a total of 362 results were obtained. Based on the title and abstract screening, 2,169 records were eliminated as not relevant. Around 486 records were examined in more detail considering the methodology and the content of the results and another 463 articles were eliminated. The total number of studies included was 23 including results from additional Google Scholar searches and hand searches.

### Thematic Synthesis of Results

**Figure 1.**
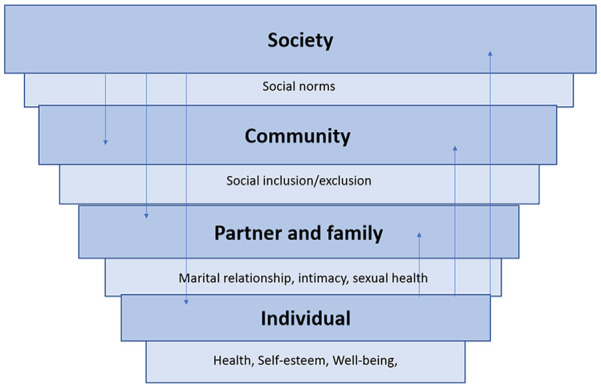
Ecological framework.

The included studies were coded by thematic areas. Recognizing that FGM can be considered to be a social norm in many high-prevalence settings, the thematic analysis was conducted separately for studies which were conducted in high-prevalence settings as opposed to among women from countries where FGM occurs but who are living in the diaspora. Once the analysis and thematic coding were completed, it was decided that the social ecological framework (McLeroy et al., 1988), which depicts the layers of influence affecting human behavior and health, was an appropriate framework for structuring the themes that emerged during the analysis (Figure 1). The ecological framework in Figure 1 provides a structure for understanding the complex effects of FGM and shows the bidirectional relationship between different layers within the framework. The synthesis of results is presented according to this framework so that societal, community, family, relationship, and individual effects of FGM are described in turn, with separate syntheses for studies within high-prevalence settings and those of diaspora populations.

**Figure 2.**
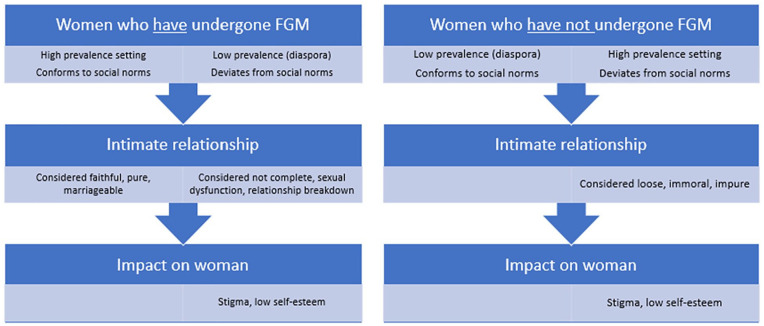
FGM status can affect women differently in high prevalence versus diaspora settings. *Note.* FGM = female genital mutilation.

The thematic analysis below shows how individual women with or without FGM in a high-prevalence setting are affected differently by the practice throughout their life-course as compared to women with or without FGM in a low-prevalence setting (see Figure 3). Whether one’s FGM status is concordant or discordant with the social norms surrounding the practice in the society and community in which one lives will determine the ways a woman’s life may be affected. Figure 3 provides one example of how FGM can affect women in different settings depending on whether she is in a high- or low-prevalence setting and depending on her FGM status. The example provided in Figure 2 relates specifically to the effect of her FGM status on her intimate relationships and how that in turn affects her body image, her identity, and her overall well-being.

### Impact at Society and Community Level

#### Negative public discourses on FGM and felt stigma

##### Diaspora in high-income context

Studies identified in the review from Sweden, the Netherlands, the United Kingdom, and the United States describe how discourses on FGM affect migrant women and men living in diaspora (Ahlberg et al., 2001; Johnsdotter et al., 2009; Jordal et al., 2018; Kahn, 2016; Parikh et al., 2018; Vloeberghs et al., 2011). Ahlberg et al. (2004) present the views of informants who felt that they often have to lie about their circumcision status to avoid stigmatization or social exclusion in Sweden (Ahlberg et al., 2001). Respondents felt that the way in which “Swedish people” talked about the practice was hurtful and made them feel inferior (i.e., “Here the uncircumcised are the majority . . . they see us as a big thing and you feel inferior”), so they were inhibited to discuss the topic.

Exploring the psycho-social well-being of cut women in diaspora, various studies report on the perceived sense of “feeling different” and shameful (Johansen, 2002; Jordal et al., 2018; Kahn, 2016; Parikh et al., 2018; Vloeberghs et al., 2011). The respondents, who desired clitoral reconstructive surgery, expressed a sense of felt stigma and inferiority that lead them to deny their FGM status in front of others. The majority of women in this study aimed to achieve “normal looking” genitalia through the operation (Jordal et al., 2018).

Reporting a similar sense of “feeling different” and a desire for completeness, Parikh et al. (2018) describe how women react when they feel stigma—some use humor to deflect unease about the topic and others completely avoid discussion about it (Parikh et al., 2018).

Johansen describes school-girls’ sense of shock and bitterness about their condition. “As if it was written on my forehead that I was different down there” (Johansen, 2002). The sense of difference affected socialization with peers such as participating in sports and relationships with boys (Johansen, 2002). Ahlberg et al. (2004) describe how school girls of Somali origin in Sweden exercise social pressure and stigmatizing behavior by “checking each other,” that is, by paying attention to the sound of urinating in the toilets. Some girls felt that negative discourses about the practice affected the relationships between those who had undergone FGM and those who had not, affecting their sense of belonging. The psychological well-being of girls who had undergone some form of FGM was negatively affected by these discourses. For example, one girl stated, “When our teacher talks about circumcision, she dramatizes as if it was a disease . . . students pity you and think you are different.” Some parents had lied to the girl and/or to the family about her FGM status to protect her. This led to uncertainty and insecurity among the girls (Ahlberg et al., 2001) and affected their sense of trust in family members.

Various studies explore migrants’ anxieties regarding pressure from relatives in their countries of origin to have their daughters undergo FGM (Alhassan et al., 2016; Isman et al., 2013; Koukoui et al., 2017; Owojuyigbe et al., 2017; Vloeberghs et al., 2011) as it reflects on their reputation and that of their daughters. In a study on African migrants in Portugal, Spain, and Italy, migrants felt pressure from their mothers-in-law back in their home country regarding the excision of their daughters. In the country of origin, women who had not gone through FGM would be marginalized and would not be able to participate in community activities, ceremonies, and decision-making. “Uncircumcised” women in Guinea Bissau were labeled “*solima*” (rude, immature, uncivilized) and *blufo* (stupid, promiscuous). Likewise, in Eritrea uncut, women are called prostitutes. Although such labels were not as extensively used in diaspora, parents were concerned about their daughters’ and their own reputation when returning home. Those who did not have their daughters cut were said to be accused of under-valuing their culture and were stigmatized back home (Alhassan et al., 2016).

Some studies address parents’ concerns regarding their daughters’ reputation and ability to control their sexual appetite if they were left uncut (Alhassan et al., 2016; Koukoui et al., 2017). Looking at migrants from FGM practising communities in Canada and in Ivory Coast, Koukoui et al. (2017) found that parents were concerned that their uncut daughters’ sexual behavior may bring shame not just to themselves and their parents but to the larger family and neighbors. They would thus be shunned and excluded (Koukoui et al., 2017).

Based on interviews with migrants from FGM practising countries living in the United States, Kahn (2016) describes how deviating from the social norm was linked to punishment and banishment by community members. Those who acted upon transgressive beliefs were expelled from the community in an act of cultural silencing

So even when you want to go against [circumcision], you don’t want to open your mouth and say it. You’ll feel scared. Because you don’t want to be lonely in society . . . Even if you don’t like it, you don’t want to say it. (Kahn, 2016)

O’Neill et al. (2017) found that this form of exclusion of women from marriage was reported particularly by older Somali male migrants in Europe when they were speaking of their country of origin. Younger Somali men and other migrants from FGM practising countries living in diaspora said that although the practice was perceived as important, they personally preferred a wife who had not been cut (O’Neill et al., 2017).

##### High-prevalence setting where FGM is a social norm

Several studies in this review showed stigmatization of women who have not undergone FGM, with implications on their marriageability. Women who had been cut were thought of as more mature, responsible, trust-worthy, and (sexually) faithful—which are all desirable traits for a bride and wife; in contrast to this, those who had not undergone FGM were not deemed marriageable, were ostracized, and publicly shamed (Abathun et al., 2016; Brown et al., 2016; Isman et al., 2013; O’Neill et al., 2017). Examples were cited of women being returned to their parents after the wedding night because the women had not gone through the practice (Abathun et al., 2016; O’Neill et al., 2017).

Shell-Duncan et al. (2018) found that with the increasing acceptability of interethnic marriages in Senegambia, it was more common for uncut women to marry into families who carry out the practice. Uncut women, however, reported their female kin’s disapproval, which was expressed through verbal abuse, harassment, and exclusion. Uncut women are commonly contemptuously labeled “*solema*” (meaning rude, ignorant, uncivilized, over-sexed, and unclean), and family members refused to eat food that they prepared and excluded them from family events such as weddings and household decision-making. Parents who do not a girl face judgments about the moral propriety of their daughters and whether they are raised properly—which reflects the social standing of the whole family within the community (Shell-Duncan et al., 2018). Similarly, Abdelshahid and Campbell (2015) showed that parents in Egypt were afraid of their daughters’ social acceptance. Uncut girls were believed to disgrace themselves through their sexual behavior and needed to be “protected” from “wrong-doing” and the humiliation they would bring to themselves if they were left uncut. It was believed that uncut girls would go “astray” and get uncontrollably aroused by anything coming close to her body (Abdelshahid & Campbell, 2015).

#### Relationships with family

##### Diaspora in high-income context

Three studies set in Sweden, the United States and the Netherlands describe women’s memories of feeling abandoned by the people close to them on the day they were cut, particularly their mothers who did not help them (Berggren et al., 2006; Kahn, 2016; Vloeberghs et al., 2011). Some expressed a sense of betrayal, anger, and frustration, and deplored that nobody had asked them and that the decision to cut had been taken by the elders (Berggren et al., 2006; Kahn, 2016; Vloeberghs et al., 2011). Studies conducted in Canada, the Netherlands, Sweden, and the United States suggest that women who had suffered from the consequences in one way or another were determined to protect their daughters from getting cut (Berggren et al., 2006; Isman et al., 2013; Kahn, 2016; Koukoui et al., 2017; Vloeberghs et al., 2011). Some expressed great relief about having left their country of origin where it was difficult to escape the practice and that they were now living in a place where their daughters were safe (Kahn, 2016; Koukoui et al., 2017; Vloeberghs et al., 2011). One way of protecting the daughters was to not leave them alone with their grandmothers who might cut them without consent of the parents (Isman et al., 2013; Johnsdotter et al., 2009; Kahn, 2016; Koukoui et al., 2017; Vloeberghs et al., 2011).

##### High-prevalence setting where FGM is a social norm

Schultz and Lien (2014) address how FGM affected relationships between mothers and daughters in the Gambia immediately after the FGM was performed in the ritual initiation context. Negative reactions described were varying degrees of pain, anxiety, disbelief, a sense of betrayal, and anger at the mother. Although the effects on the mother–daughter relationship seemed mostly short-term, as Schulz and Lien suggest, several women experienced emotional challenges in their relationships with their mothers once they lived in exile and were exposed to arguments against FGM. Other women reported pride and described initiation as a bonding experience endowing them with status within the community (Schultz & Lien, 2014).

### Impact on Intimate Relationships

#### Sexual intimacy and marital relationships

##### Diaspora in high-income context

Multiple studies documented the difficulties posed by FGM on intimate relationships and sexual health. Studies set in Belgium, the Netherlands, the United Kingdom, and the United States present the experiences of women and men whose intimate relationships and sexuality are affected by FGM (Johansen, 2002; Kahn, 2016; O’Neill et al., 2017; Vloeberghs et al., 2011). Vloeberghs et al. (2011) and Johansen (2002) present women’s accounts of painful sex due to their FGM, regrets about not experiencing any pleasure or about sex being boring. Johansen describes how sex during marriage was perceived to be a duty for a wife, regardless of how painful (Johansen, 2002). Vloeberghs et al. (2011) describe how some said they pretended to enjoy it to please their husband, or that they came up with excuses not to have sex when their partners showed interest.

In a study looking at African migrants from FGM practising communities in Belgium, the Netherlands, and the United Kingdom, O’Neill et al. (2017) found that although some women reported no sexual problems linked to FGM, other women complained that they did not enjoy sex very much but did it to please their partners or because they perceived it to be a duty (O’Neill et al. 2017). Similarly, some men had not noticed any difference during sexual relations between women who had been through the practice and those who had not. However, others felt emotionally affected by their partners’ lack of pleasure and difficulty in having orgasms. Some respondents stated that their divorces were caused by the ways that FGM had affected their intimate relationships. Many older Somali and Ethiopian men in the study suggested that although casual sexual relations with uncut women were enjoyable, uncut women were not for marriage and that such relationships would not last. Their younger counterparts residing in Europe who participated in the study felt that love was a more important criteria for marriage than FGM status. A study based in Norway found that uncut women were preferred as marriage partners among Norwegian Somalis (Gele et al., 2012).

Ahlberg et al. (2004) present Swedish Somali’s conflicting views on different types of FGM, virginity, and trust. Some men believed that infibulation was not proof of a woman’s virginity because even women who had a child could have been re-infibulated and married as virgins afterwards. Women on the other hand thought that men were more suspicious of un-infibulated women, who had undergone Type I, II, or IV FGM, and cases were reported of un-infibulated virgin brides being sent back to their parents because the husband and in-laws doubted the girl’s virginity status (Ahlberg et al., 2001). Two studies show how sexual pain had a negative impact on experiences in finding a committed partner (Kahn, 2016; Vloeberghs et al., 2011). Kahn (2016) presents women’s experiences of disliking sex and having pain during intercourse, which led to a chain of short-term relationships with men rather than long-lasting committed and supportive relationships. Other research set in Sweden suggests that the diminished enjoyment of sex is a result of cultural discourses according to which sex, for women, should not be enjoyable because it is dirty. Thus their reduced pleasure may also be linked to the embodiment of such cultural discourses (Johnsdotter et al., 2009).

##### High-prevalence setting where FGM is a social norm

Looking at the effects of FGM on sexuality in southern Ethiopia, Battle et al. (2017) report on men and women’s descriptions of pain and pleasure among newly wedded couples. Women with type-III FGM described traumatic experiences of first sex including extreme pain, bleeding, fear, fainting, becoming ill, and fleeing forced first sex. The husbands of infibulated women also reported pain and abrasions on their penis after prolonged unsuccessful attempts at penetration. This painful and traumatic process was, however, culturally endorsed and positively associated with masculinity. Men described this experience with pleasure and pride, which was particularly linked to a sense of feeling reassured that no other man had ever penetrated their wife. In this study, this process is thus believed to create a bond of trust and confidence between man and wife—a reassurance that she would remain faithful to him (Battle et al., 2017).

Battle et al. (2017) also describe the breakdown of relationships between women and men as being linked to women’s dislike of sex, pain, and trauma. Three major strategies for avoiding sex emerged in their research in southern Ethiopia; these included direct refusal of sex, physical, and psychological distancing, and the promotion of other sex partners. For instance, leaving a husband’s bed in the middle of the night, sleeping with children and pretending to be sick were mentioned as ways of avoiding sexual contact with one’s husband. Battle describes that violence toward a wife was a culturally endorsed response to resistance to sexual intercourse among her Somali study participants in Ethiopia.

Men also avoided sex in marriage as a result of sexual difficulties with their wives but used different tactics, such as engaging in extra-marital affairs, polygamy, or divorce. Commonly mentioned reasons for leaving a wife were related to her “bad behavior”—such as fighting, being too demanding, and infertility. The distinct marriage patterns of polygamy and divorce suggest dissatisfaction and tensions in marriages to infibulated women (Battle et al., 2017). A study set in Southern Nigeria also shows that although FGM contributes to a woman’s respectability and reputation as a wife, almost all men with excised wives admitted to regularly having sex outside marriage with multiple partners. Nevertheless sex with their excised wives was perceived as a duty, and they continued to have sexual relations with their wives (Owojuyigbe et al., 2017).

Studies set in Egypt, Kenya, Nigeria, and Ethiopia have explored men’s feelings about their wives’ FGM status. A study in Egypt explored how women and men experience conflict as a result of lack of sexual satisfaction and pain (Abdelshahid & Campbell, 2015). Some women stated that it was not possible for them to share their feelings of rejection, the physical pain during intercourse, or arguments within the couple with other community members due to the private nature of the issue. It was considered taboo for women to speak of sexuality. Although married men regretted their wives’ lack of sexual response and desire, they felt reassured that their wives did not enjoy sex enough to go and seek it elsewhere. That is,

When I sleep with my wife, I’d like her to have a desire for me . . . lack of response, of course, doesn’t appeal to any man . . . it doesn’t satisfy any man . . . but when I feel that my wife has no sexual drives, I feel reassured, but, when we sleep together I feel bad because she doesn’t have a desire for me. (Abdelshahid & Campbell, 2015)

Various studies show that men’s feelings about the effect of the FGM on women’s sexuality are ambivalent (Abathun et al., 2016; Abdelshahid & Campbell, 2015; Brown et al., 2016). Studies set in Ethiopia and in Kenya describe how young men felt enormous social pressure to marry women with FGM but feared being socially outcast and denied social status and political influence in local governance structures. Nevertheless, these young men did not embrace the idea that marriage to a cut woman as they were aware of the negative health consequences of the practice and felt that they should oppose this custom in spite of their family’s expectations; however, this was not always easy to negotiate (Abathun et al., 2016; Brown et al., 2016).

Graamans et al.’s (2018) study on Maasai and Samburu in Kenya suggests that negative conceptions of women’s sexuality have given way to positive associations regarding women’s desire of wanting to love their husbands, their indulgence in pleasure, and the “sweetness of love,” which were thought to be absent in women with FGM. It was suggested that this might affect the intimacy which bonds husband and wife and holds the family together. Having undergone FGM thus led to insecurities and anxieties about a husband looking for new partners and wives (Graamans et al. 2018).

### Impact at Individual Level

#### Self-esteem, anxiety, trauma, and other psychological consequences

##### Diaspora setting in high-income context

In a study among women who were seeking clitoral re-constructive surgery in Sweden, Jordal et al. (2018) explored women’s conceptions of how FGM affected their sex-life and intimate relationships. They found that they perceived their inability to enjoy sex as a handicap because they were living within a context in which women’s sexual pleasure and indeed mutual pleasure was viewed as important for closeness and intimacy. Although some of them were able to enjoy sex and reach orgasm with the right partner or when masturbating alone, there was a general sense of inadequacy among the informants who perceived their genitalia as ugly and dysfunctional, which caused shame and uneasiness in sexual relations as well as negative effects on their self-esteem. This negative self-image led some of them to avoid certain sexual practices associated with pleasure or avoid sex and intimacy altogether. These women hoped that reconstructive surgery would improve their self-esteem and likewise their sexual relations (Jordal et al., 2018).

Studies set in the Netherlands and the United States described how anxiety, trauma, and anger related to FGM affected women’s social lives, behavior, and relationships with others. Vloeberghs et al. (2011) describe the experiences of women who were troubled by unpleasant memories when confronted with particular situations or reminders, such as razor blades, knives, blood, sexual intercourse, including on their wedding night, and child-birth (Kahn, 2016; Vloeberghs et al., 2011). These reminders could trigger a range of profound emotional reactions such as crying, feeling “sick and like throwing-up” (Kahn, 2016; Vloeberghs et al., 2011) as well as a sense of powerlessness (Kahn, 2016).

In response to negative discourses about FGM or personal experiences where women felt stigmatized, studies describe avoidance and deflection (Parikh et al., 2018; Vloeberghs et al., 2011) as well as anger about media images that depict the practice as barbaric and incite pity among the audience (Johnsdotter et al., 2009). Studies suggested that women do not want to be pitied or seen as victims because of the practice (Jordal et al., 2018; Vloeberghs et al., 2011). Vloeberghs et al. (2011) also describe their respondents’ anger and frustration when they realize that FGM is not necessary, that it was not a religious requirement, and that not all women were cut. Anger was provoked by a sense of injustice that they had been cut and others had not and sometimes anger with relatives who allowed the practice to happen. Others expressed their anger more generally toward what they called the “backward” traditions and “ignorance” that made women perform the practice (Vloeberghs et al., 2011).

##### High-prevalence setting where FGM is a social norm

There is lack of qualitative research describing psychological consequences such as posttraumatic stress disorder (PTSD), anxiety, and low self-esteem related to FGM in high-prevalence settings. Multiple studies comparing women with and without FGM on clinical outcomes have found that women with FGM have higher rates of these psychological complications, such as depression, anxiety, and posttraumatic stress disorder, as compared to women without FGM (M. R. Ahmed et al., 2017; Behrendt & Moritz, 2005; Kizilhan et al., 2017; Köbach et al., 2018).

These studies did not meet the inclusion criteria of this review, which summarizes the qualitative research in this area and complements these clinical findings.

### Impact of FGM Status on Women’s Interactions Within Institutional Context

The ecological framework (Figure 2) depicts the bidirectional nature of these experiences and how women’s FGM status can impact on her intimate relationships and potentially her marital status. These aspects in turn have an impact on how she interacts within larger societal contexts and how she is viewed by her community which in turn can affect her self-esteem and well-being. Social norms and societal discourses directly affect how women perceive their body and how they relate and interact with others.

### Health Care Settings

Some studies show that a woman’s FGM status can affect her health-seeking behavior and her experiences when accessing certain health services. Three studies from European countries explored stigma experienced in health care settings, particularly during pregnancy and postpartum. In one study, women reported feeling embarrassed during examinations performed by health professionals (Pastor-Bravo et al., 2018). Berggren et al. (2006) and Vloeberghs et al. (2011) describe how women felt stared at by health professionals, and sometimes they perceived disgust or a lack of respect shown to them, which made them feel ashamed (Berggren et al., 2006; Vloeberghs et al., 2011). Earlier studies describe Somalis’ experiences of health professionals’ incompetence during labor and how to deinfibulate them, which caused unnecessary problems during labor and delivery (Ahlberg et al., 2001; Berggren et al., 2006). When health care providers did not discuss FGM status during prenatal consultations, women sometimes thought that the health professionals were aware of how to conduct deinfibulation (Berggren et al., 2006). Language problems were also said to be an issue during delivery, so that women could not discuss the deinfibulation process with their health care provider (Ahlberg et al., 2001). The lack of awareness and stigmatizing attitudes held by health care providers–created barriers in service delivery, which have been shown to have negative effects on health seeking.

### Asylum Procedures and Safeguarding Agencies

Two U.K.-based studies looked at how the topic of FGM was addressed in asylum procedures and how governmental agencies attended to women because they were from FGM practising countries (Kea & Roberts-Holmes, 2013). Parikh et al. (2018) describe how women from FGM practising countries in the United Kingdom feel stigmatized by social services and safeguarding agencies even if they are personally opposed to the practice.

Kea and Roberts-Holmes (2013) show how women with FGM take on “victim identities” as a result of the profiling during the asylum procedure, which forces them to disclose and retell traumatic and intimate stories of violence which objectify them as victims. The process of reproducing accounts of one’s home, one’s ethnic identity and one’s family members as backward and barbaric—in line with the social norms and cultural context of the country where asylum is sought—can be a confusing and psychologically damaging one (Kea & Roberts-Holmes, 2013).

## Discussion

This review provides a framework for understanding the many ways in which women and girls with FGM experience adverse effects that affect their participation in society, the way society views them, their sense of identity, and their well-being. These consequences are often not well documented and not well addressed in programs and during encounters within the health care system. Whether in high-prevalence settings or in diaspora, FGM status itself does not define a woman’s identity but rather is one aspect of her identity that is closely linked with social norms.

Deviating from social norms can affect women’s lives, sense of well-being, and self-esteem in different ways. Figure 2 shows how uncut women in high-prevalence settings may be more vulnerable to stigma, as they may be labeled as being loose, immoral, or impure, which would affect their self-esteem. On the other hand, a cut woman in a diaspora setting may feel stigmatized as a result of being labeled “sexually handicapped” and incomplete, which would affect her psycho-social well-being and self-esteem.

The findings of this review complement existing literature that has demonstrated the effects of FGM on sexual functioning and sexual health, including dyspareunia, reduced sexual satisfaction, and reduced sexual desire (Berg et al., 2010). This review of qualitative research does not explore whether FGM increases the risk of sexual health problems, but rather how reduced sexual satisfaction and other symptoms that are classified as “sexual dysfunction” may affect interpersonal and relationship dynamics in both high-prevalence settings and diaspora communities (Abdelshahid & Campbell, 2015; Ahlberg et al., 2001; Battle et al., 2017; Brown et al., 2016; Graamans et al., 2018; Kahn, 2016; Jordal et al., 2018; O’Neill et al., 2017; Owojuyigbe et al., 2017; Vloeberghs et al., 2011). Difficulty in negotiating safe sex practices (Chai et al., 2017), intimate partner violence, including forced sexual intercourse can also be the result of these dynamics, but additional research is needed in these areas (Abdelshahid & Campbell, 2015; Battle et al., 2017; Esho et al., 2017; Vloeberghs et al., 2011).

This review has shown that in settings where FGM is a social norm, the negative connotations of uncut women as oversexed, unfaithful, and incapable of regulating their sexual desires continues to have a significant impact on the perpetuation of the practice (Abathun et al., 2016; Abdelshahid & Campbell, 2015; Battle et al., 2017; Shell-Duncan et al., 2018). Studies in diaspora show that migrants are often concerned about their uncut daughters’ reputation and that not cutting them might affect their respectability and social status in their communities back home (Alhassan et al., 2016; Kahn, 2016; Koukoui et al., 2017). Thus, in many cases, the psycho-social consequences of not practising can affect women’s psycho-social well-being in places where the practice is a social norm.

In general, the impact of FGM status on women’s engagement with the larger society was more salient for migrants living in countries where the practice is not a social norm. In response to the growing influx of migrants from FGM practising countries in Europe and North America, several studies describe the experiences of women with FGM during asylum procedures (Kahn, 2016; Kea & Roberts-Holmes 2013; Parikh et al., 2018); at school and in their everyday lives (Ahlberg et al., 2001; Johansen, 2002), as well as in health care settings (Ahlberg et al., 2001; Berggren et al., 2006; Johansen, 2002; Vloeberghs et al., 2011). Feeling judged or stigmatized during health care encounters may adversely affect women’s health care decision-making and their attendance at routine health visits and accessing of necessary health-interventions (Evans et al., 2019; Hamid et al., 2018); however, to date, there is limited evidence and more research on this is needed. Although the themes that emerged in the review are stratified by high-prevalence settings and diaspora populations, this does not imply that all women living in these environments are a homogeneous group that experience FGM status in the same way. The diversity of experience of particular subgroups of women may not be reflected in these generalized patterns.

### Limitations

While efforts were made to include a wide range of themes, some studies relating to psycho-social well-being may have been unintentionally missed in the published or gray literature limiting the range of themes identified in this review. Measurement of well-being is in fact limited, partially because these are difficult concepts to quantify (Healey-Ogden & Austin, 2011; Morse, 2006). Understanding the psycho-social consequences of FGM requires a range of research methods, both quantitative, and qualitative. This review focuses on the qualitative literature and is intended to complement the quantitative findings from a related analysis of studies that operationalize and measure clinical outcomes of women’s health, including psychological and sexual health outcomes among women with FGM as compared to those who have not undergone FGM (Pallitto et al., Forthcoming; Tordup et al., Forthcoming). Evidence of an increased risk of adverse outcomes among women who have undergone FGM provides an answer to one question, while qualitative research on this topic answers different complementary questions by contextualizing quantitative results. For example, qualitative research provides perspectives of experience of psycho-social outcomes, such as stigma and how such experiences create further barriers for women in their social environment. Despite the measurement limitations, the findings presented here are an important aspect of understanding the psycho-social impact of the practice and of compiling evidence of the larger societal and psychological consequences beyond quantifiable health risks.

## Conclusion

While the international community remains committed to the abandonment of FGM, it is important to recognize the complex interactions between women and their families and communities. Understanding the ways that social norms affect women through various mechanisms and across different layers in the ecological framework is an important step in ensuring that women are treated respectfully and that their rights are not violated (Figure 2).

## Supplemental Material

sj-pdf-1-qhr-10.1177_10497323211001862 – Supplemental material for The Consequences of Female Genital Mutilation on Psycho-Social Well-Being: A Systematic Review of Qualitative Research Click here for additional data file. Supplemental material, sj-pdf-1-qhr-10.1177_10497323211001862 for The Consequences of Female Genital Mutilation on Psycho-Social Well-Being: A Systematic Review of Qualitative Research by Sarah O’Neill and Christina Pallitto in Qualitative Health Research
