# Supplementary material for: The Consequences of Female Genital Mutilation on Psycho-Social Well-Being: A Systematic Review of Qualitative Research
Source: Qual Health Res. 2021 Jun 8;31(9):1738–50. doi: 10.1177/10497323211001862 (PMC8438768; doi:10.1177/10497323211001862)
Supplement: sj-pdf-1-qhr-10.1177_10497323211001862 – Supplemental material for The Consequences of Female Genital Mutilation on Psycho-Social Well-Being: A Systematic Review of Qualitative Research [file sj-pdf-1-qhr-10.1177_10497323211001862.pdf]

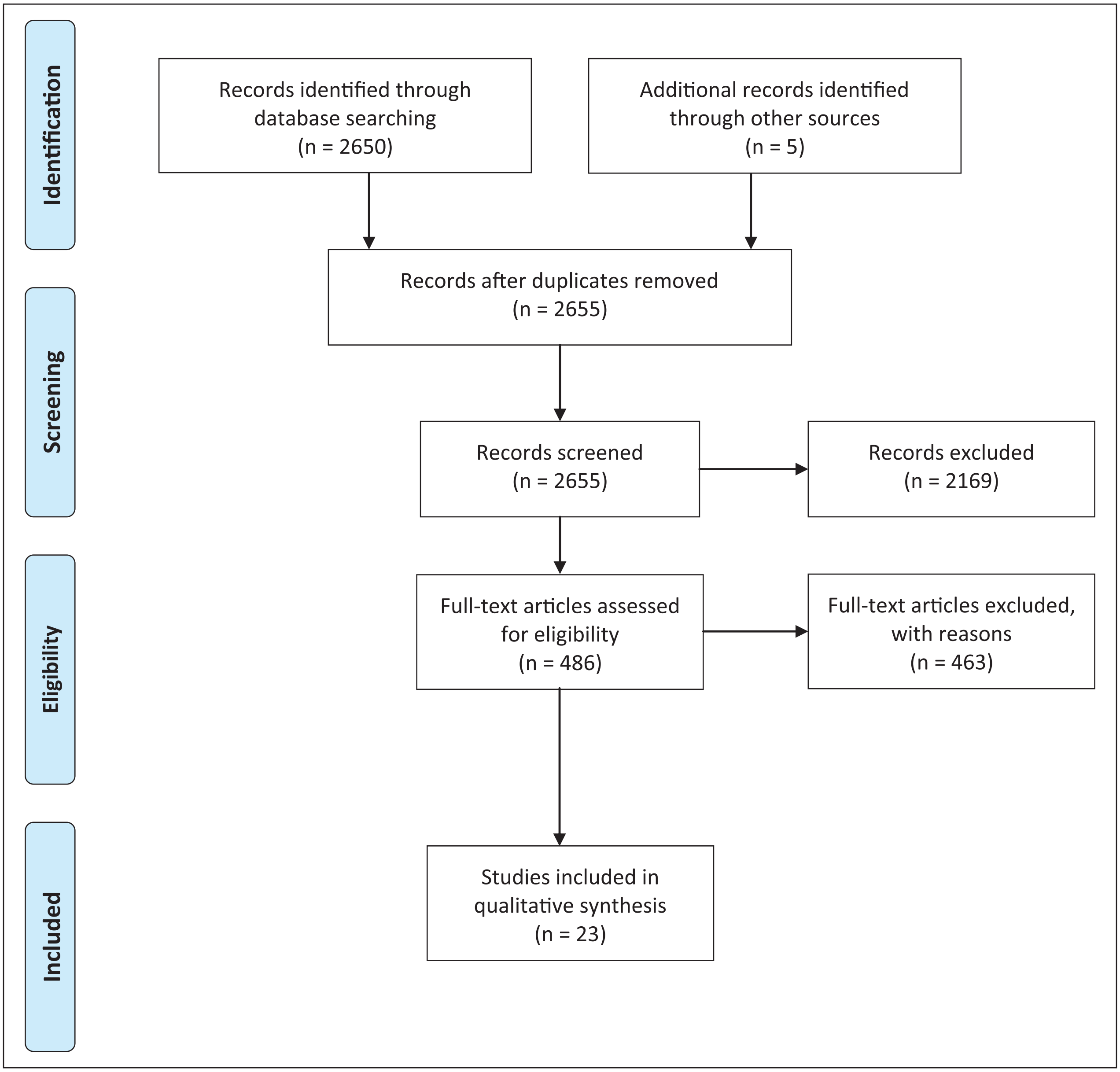

Figure 1. PRISMA 2009 flow diagram.
Source. Moher et al. (2009).

Table 1. Summary of included studies

No.	Authors	Year	Title	Journal	Study setting	Ethnicity	Prevalence	Types of FGM among study participants	Total sample included	Methods
1	Abathun AD, Sundby J, Gele AA.	2016	Attitude Toward Female Genital Mutilation Among Somali and Harari People, Eastern Ethiopia.	International Journal of Women's Health 8, 557–569	Ethiopia	Somali and Harari	High-prevalence setting	Types I, II, III	64	8 FGDs
2	Abdelshahid A, Campbell C	2015	"Should I Circumcise My Daughter?" Exploring Diversity and Ambivalence in Egyptian Parents' Social Representations of Female Circumcision	Journal of Community & Applied Social Psychology 25(1), 49–65	Egypt	Egyptian	High-prevalence setting	not mentioned	16	16 IDIs
3	Alhassan, Yussif Nagumse; Barrett, Hazel; Brown, Katherine E.; Kwah, Kayleigh	2015	Belief Systems Enforcing Female Genital Mutilation in Europe	International Journal of Human Rights in Healthcare 9(1), 29–40.	Portugal, Spain, Italy	Guinea-Bissau, Eritrea	Low-prevalence setting <1%)	types I, II, III	Italy=96, Portugal =86, Spain=96	24 FGDs 70 IDIs
4	Ahlberg, BM; Krantz, I; Lindmark, G; Warsame, M	2004	"It's only a tradition": Making Sense of Eradication Interventions and the Persistence of Female "Circumcision" Within a Swedish Context	Reproductive Health Matters 9(17), 26–36.	Sweden	Somali	Low-prevalence setting (<1%)	not mentioned	110	FGDs and IDIs
5	Battle, J.D., Hennink, M.M., Yount, K.M.	2017	Influence of Female Genital Cutting on Sexual Experience in Southern Ethiopia	International Journal of Sexual Health, 29(2), 173–186.	Ethiopia	Somali, Harari	High-prevalence setting	Types I, II, III	49	IDIs
6	Berggren V.; Bergström S, Edberg A. K.	2006	Being Different and Vulnerable: Experiences of Immigrant African Women Who Have Been Circumcised and Sought Maternity Care in Sweden	African Journal of Reproductive Health 10(2).	Sweden	Somalia, Sudan, Eritrea, Sweden	Low-prevalence setting (<1%)	not mentioned	22	IDIs
7	Brown E, Mwangi-Powell F, Jerotich M, le May V.	2016	Female Genital Mutilation in Kenya: Are Young Men Allies in Social Change Programs?	Reproductive Health Matters 24(47), 118–125.	Kenya	Pokot	High-prevalence setting	Type III	72	IDIs
8	Gele AA, Kumar B, Hjelde KH, Sundby J.	2012	Attitudes Toward Female Circumcision Among Somali Immigrants in Oslo: A Qualitative Study	International Journal of Women's Health 4, 7–17.	Norway	Somali	Low-prevalence setting (<1%)	Type III	36-40	4FGDs and 16 IDIs
9	Graamans E, Ofware P, Nguura P, Smet E, Ten Have W.	2018	Understanding Different Positions on Female Genital Cutting Among Maasai and Samburu Communities in Kenya: A Cultural Psychological Perspective	Culture, Health and Sexuality 1058, 1–16.	Kenya	Massai, Samburu	Not mentioned	not mentioned	72	IDIs
10	Isman E, Ekéus C, Berggren V.	2013	Perceptions and Experiences Of Female Genital Mutilation After Immigration to Sweden: An Explorative Study	Sexual and Reproductive Healthcare 4(3), 93–98.	Sweden	Ethiopia, Somalia, Djibouti, Eritrea	Low-prevalence setting (below <1%)	not mentioned	8	IDIs
11	Johansen, E.R.	2002	Pain as a Counterpoint to Culture: Toward an Analysis of Pain Associated With Infibulation Among Somali Migrants in Norway	Medical Anthropology Quarterly 16(3)	Norway	Somali	Low-prevalence setting (below <1%)	98% among Somalis in Norway	30	IDIs, participant observation
12	Johnsdotter, S., Moussa, K., Carlbom, A., Aregai, R., Essen, B.	2009	Never My Daughters: A Qualitative Study Regarding Attitude Change Toward Female Genital Cutting Among Ethiopian and Eritrean Families in Sweden	Health Care for Women International 30(1–2), 114–130.	Sweden	Ethiopian and Eritrean	Low-prevalence setting (<1%)	not mentioned	33	IDIs

(continued)

Table 1. (continued)

No.	Authors	Year	Title	Journal	Study setting	Ethnicity	Prevalence	Types of FGM among study participants	Total sample included	Methods
13	Jordal M, Griffin G, Sigurjonsson H.	2018	"I want what every other woman has": Reasons for Wanting Clitoral Reconstructive Surgery After Female Genital Cutting—A Qualitative Study From Sweden	Culture, Health and Sexuality 0(0), 1–16.	Sweden	Somalia, Eritrea, The Gambia, Sierra Leone, Iraq (Kurdistan)	Low-prevalence setting (<1%)	not mentioned	17	IDIs
14	Kahn, S	2016	You See, One Day They Cut: The Evolution, Expression, and Consequences of Resistance for Women Who Oppose Female Genital Cutting	Journal of Human Behavior in the Social Environment 26(7–8), 622–635.	United States	Guinea, Burkina Faso, The Gambia, Chad	Low-prevalence setting (<1%)	not mentioned	14	*IDIs*
15	Kea, P. J.; Roberts-Holmes, G.	2013	Producing Victim Identities: Female Genital Mutilation and the Politics of Asylum Claims in the United Kingdom	Identities 20(1), 96–113.	The United Kingdom	Mandinka, Fula	Low-prevalence setting (<1%)	not mentioned	12	IDIs
16	Koukoui S, Hassan G, Guzder J.	2017	The Mothering Experience of Women With FGM/C Raising 'Uncut' Daughters, in Ivory Coast and in Canada	Reproductive Health 14(1), 1–11.	Canada	Somalia, Djibouti, Ethiopia, Mali, Guinea, Egypt	Low-prevalence setting (<1%)	not mentioned	15	IDIs
17	O'Neill S., Dubourg D., Florquin S., Bos M., Zewolde S., Richard F.	2017	"Men Have a Role to Play but They Don't Play it": A Mixed Methods Study Exploring Men's Involvement in Female Genital Mutilation in Belgium, the Netherlands and the U.K. Report	MSO Report, Daphne EU	Belgium, Netherlands, the United Kingdom	Egyptian, Ethiopia, Somali, Sudanese, Guinean, Sierra-Leone	Low-prevalence setting (<1%)	not mentioned	135	60 IDIs and 9 FGDs
18	Owojuyigbe M., Bolorunduro M.-E., Busari D.	2017	Female Genital Mutilation as Sexual Disability: Perceptions of Women and Their Spouses in Akure, Ondo State, Nigeria	Reproductive Health Matters 25(50), 80–91.	Nigeria	Yoruba	High-prevalence setting	not mentioned	22	IDIs
19	Parikh N, Saruchera Y, Liao LM	2018	It is a Problem and it is not a Problem: Dilemmatic Talk of the Psychological Effects of Female Genital Cutting	Journal of Health Psychology 2018 https://doi.org/10.1177/1359105318781904	The United Kingdom	Sudan, Somalia, Somaliland, Gambia, Nigeria, Sierra Leone and Saudi Arabia	Low-prevalence setting (<1%)	Types I, II, III, IV	13	IDIs
20	Pastor-Bravo MDM, Almansa-Martinez P, Jiménez-Ruiz I.	2018	Living With Mutilation: A Qualitative Study on the Consequences of Female Genital Mutilation in Women's Health and the Healthcare System in Spain	Midwifery 66, 119–126.	Spain	Jola, Mandinka (Senegal), Igbo (Nigeria)	Low-prevalence setting (<1%)	Types I, II	14	IDIs
21	Schultz, J.H and Lien, Inger-Liese	2014	Cultural Protection Against Traumatic Stress: Traditional Support of Children Exposed to the Ritual of Female Genital Cutting	International Journal of Women's Health 6, 207–219	The Gambia	ethnicity not mentioned	High-prevalence setting	Types I, II, III, IV	33	IDIs
22	Shell-Duncan B, Moreau A, Wander K, Smith S.	2018	The Role of Older Women in Contesting Norms associated with female genital mutilation/cutting in Senegambia: A factorial focus group analysis	PLOS ONE 13(7), 1–19.	Senegambia	Mandinka, Fula, Wolof, Tilabonka	High-prevalence setting	Types I, II, III	approx. 105	15 FGDs
23	Vloeberghs E., Knipscheer J., van der Kwaak A., Naleie, Z., van den Muijsenbergh, M.	2011	Veiled pain—Psychological, Social and Relational Consequences of Female Genital Mutilation Among Immigrant Women in the Netherlands	Pharos	Netherlands	Somalia, Sudan, Eritrea, Ethiopia and Sierra Leone	Low-prevalence setting (<1%)	Types I, II, III, IV	66	52 IDIs and 2 FGDs

Note. FGM = female genital mutilation.
